# Social Media Use and Subsequent E-Cigarette Susceptibility, Initiation, and Continued Use Among US Adolescents

**DOI:** 10.5888/pcd20.220415

**Published:** 2023-09-07

**Authors:** Juhan Lee, Suchitra Krishnan-Sarin, Grace Kong

**Affiliations:** 1 Department of Psychiatry, Yale School of Medicine, New Haven, Connecticut

## Abstract

**Introduction:**

Social media has a large amount of e-cigarette content. Little is known about the associations between social media use and a wide range of e-cigarette use behaviors, including susceptibility, initiation, and continued use. We analyzed national data on US adolescents to assess these associations.

**Methods:**

We used data on adolescents participating in the Population Assessment of Tobacco and Health (PATH) Study Wave 4 (2016–2018) and Wave 5 (2018–2019). We conducted 2 models: 1) a multinomial logistic regression on e-cigarette use susceptibility and use behaviors at Wave 5 by social media use at Wave 4 among adolescents who never used e-cigarettes at Wave 4 and 2) a binomial logistic regression on current e-cigarette use at Wave 5 by social media use at Wave 4 among adolescents who ever used e-cigarettes at Wave 4.

**Results:**

Among adolescents who never used e-cigarettes at Wave 4 (n = 7,872), daily social media use (vs never) was associated with a higher likelihood of being susceptible to e-cigarette use (adjusted odds ratio [aOR] =1.46; 95% CI, 1.20–1.78), past e-cigarette use (aOR = 3.55; 95% CI, 2.49–5.06), and current e-cigarette use (aOR = 3.45; 95% CI, 2.38–5.02) at Wave 5. Among adolescents who ever used e-cigarettes at Wave 4 (n = 794), we found no significant association between social media use at Wave 4 and continued e-cigarette use at Wave 5.

**Conclusion:**

Our study found that social media use is associated with subsequent susceptibility to e-cigarette use and initiation but not with continued use of e-cigarettes among US adolescents. These findings suggest that understanding and addressing the association between social media and e-cigarette use is critical.

**Summary**

**What is already known on this topic?**

The prevalence of social media use among adolescents is high, and social media has extensive e-cigarette content.

**What is added by this report?**

Use of social media among adolescents is associated with being susceptible to and initiating e-cigarette use in subsequent years.

**What are the implications for public health practice?**

Preventing adolescent exposure to e-cigarette content on social media is important.

## Introduction

In 2022, 95% of adolescents aged 13 to 17 years used social media (1). Social media platforms have extensive e-cigarette–related content (2). This content may be user-generated, such as a person posting about e-cigarettes to their own social network, or the industry posting marketing content with themes that appeal to adolescents (eg, vape tricks) (3–5). In general, e-cigarettes are positively portrayed on social media as “glamourous,” “healthy,” and “safe” (6).

Previous longitudinal studies showed that social media use behaviors, such as exposure to and engagement with tobacco-related content on social media, are associated with e-cigarette initiation among adolescents (7,8). Nonetheless, understanding of whether social media use is associated with the full spectrum of e-cigarette use behaviors among adolescents, such as susceptibility to e-cigarette use and continued use of e-cigarettes, is limited. Understanding susceptibility to use is important because it is an established predictor of e-cigarette use initiation among adolescents (9). Examining continued use of e-cigarettes among adolescents who are already using these products is also important because progression to regular use can lead to nicotine addiction and exposure to other toxicants and chemicals (10,11). Thus, understanding the association between susceptibility to e-cigarette use and continued use of e-cigarettes and social media is critical to fully understanding a wide range of adolescent e-cigarette use behaviors.

We used a nationally representative sample of adolescents in the US to examine longitudinal associations between social media use and susceptibility to, initiation of, and continued use of e-cigarettes. We hypothesized that more frequent social media use would be associated with higher levels of susceptibility to e-cigarette use, initiation of, and continued use of e-cigarettes.

## Methods

We used data on adolescents participating in Wave 4 (2016–2018) and Wave 5 (2018–2019) of the Population Assessment of Tobacco and Health (PATH) Study, a nationally representative longitudinal panel survey data set in the US (12). The PATH Study uses multistage stratified sampling; thus, responses in the adolescent data set represent the US population of adolescents aged 13 to 17 years. We used 2 analytic samples of adolescent respondents who completed surveys at both Wave 4 and Wave 5: 1) adolescents who never used e-cigarettes at Wave 4 (n = 7,872), to examine the likelihood of susceptibility to and initiation of e-cigarette use at Wave 5; and 2) adolescents who ever used e-cigarettes at Wave 4 (n = 794), to examine the likelihood of continued use of e-cigarettes at Wave 5. Because respondents aged 17 years at Wave 4 moved to an adult survey at Wave 5, we did not include them in our analysis.

### Measures

#### E-cigarette susceptibility, initiation, and continued use at Wave 5

For e-cigarette susceptibility at Wave 5, we used items assessing intention and willingness to use e-cigarettes (“Do you think you might try using e-cigarettes soon?” and “If one of your best friends were to offer you e-cigarettes, would you use it?”) (13). Both questions had the following response options: definitely not, probably not, probably yes, and definitely yes. When respondents reported “definitely not” to both questions, we categorized them as “nonsusceptible never-use” and others as “susceptible never-use” (9,13,14). For actual e-cigarette use behaviors at Wave 5, we used 2 variables: ever use and current (past 30 days) use of e-cigarettes. When respondents reported ever using e-cigarettes but not currently using e-cigarettes at Wave 5, we categorized them as past users. When respondents reported ever using e-cigarettes and using e-cigarettes in the past 30 days at Wave 5, we categorized them as current users.

We then created a 4-level outcome variable as follows: 0 = did not initiate and non-susceptible (nonsusceptible never-use); 1 = did not initiate but susceptible (susceptible never-use); 2 = initiated but did not currently use (past use); and 3 = initiated and currently used e-cigarettes (current use) at Wave 5 (Table 1).

**Table 1:** Model Description for Outcomes on E-Cigarette Use Among Adolescents at Wave 5 (2018–2019) and Analytic Sample at Wave 4 (2016–2018), PATH Study

Wave 4 e-cigarette use	Wave 5 e-cigarette use			
	Nonsusceptible never-use^a^	Susceptible never-use^a^	Ever use but not current use	Current use
Never user (analytic sample 1)	Reference	1 = Never but susceptible	2 = Past use	3 = Current use
Ever user (analytic sample 2)	—	—	0 = Discontinued use (reference)	1 = Continued use

Abbreviation: PATH, Population Assessment of Tobacco and Health.

a Susceptibility was categorized on the basis of “intention to use” (“Do you think you might try using e-cigarettes soon?”) and “willingness of e-cigarette use” (“If one of your best friends were to offer you e-cigarettes, would you use it?”). Only when respondents reported “definitely not” to both questions were they categorized as “nonsusceptible never-use”; others were categorized as “susceptible never-use.”

#### Social media use at Wave 4

For social media use at Wave 4, respondents were asked if they had a social media account. The survey item was as follows: “Sometimes people use the internet to connect with other people online through social networks like Facebook, Google Plus, YouTube, LinkedIn, Twitter, Tumblr, Instagram, Pinterest, or Snapchat. This is often called social media. Do you have a social media account?” If the respondent reported having a social media account, the survey asked about the frequency of social media use: “About how often do you visit your social media account?” Response options were “never,” “less often [than every few weeks],” “every few weeks,” “1–2 days a week,” “3–5 days a week,” “about once a day,” and “more than once a day.” We categorized respondents who did not have a social media account and respondents who had a social media account but never visited social media as never-users and created a 3-level predictor variable coded as 0 = never; 1 = nondaily (ie, “less often [than every few weeks],” “every few weeks,” “1–2 days a week,” “3–5 days a week”); and 2 = daily social media use (ie, “about once a day” and “more than once a day”) (15,16).

### Covariates

The covariates at Wave 4 included age (12–14 or 15–16), sex (male or female), ethnicity (non-Hispanic or Hispanic), race (White, Black, or Other [American Indian or Alaska Native, Asian Indian, Asian, Native Hawaiian, and Pacific Islander]), parental education (less than high school graduate, GED [General Educational Development], high school graduate, some college (no degree) or associates degree, bachelor’s degree, or advanced degree; annual household income (<$10,000, $10,000–$24,999, $25,000–$49,999, $50,000–$99,999, or ≥$100,000), parental e-cigarette use (no or yes), peer e-cigarette use (no or yes), e-cigarette use susceptibility at Wave 4 (only in Model 1), and current use of other drugs (other tobacco products, alcohol, cannabis, and illicit drugs).

### Statistical analyses

We conducted descriptive analyses to examine the bivariate associations between predictors at Wave 4 and outcomes at Wave 5. We further conducted 1) multinomial logistic regression analysis on e-cigarette use susceptibility and use behaviors at Wave 5 by social media use at Wave 4 among respondents who never used e-cigarettes at Wave 4 (Model 1) and 2) a binomial logistic regression model on e-cigarette use in the past 30 days at Wave 5 by social media use at Wave 4 among respondents who ever used e-cigarettes at Wave 4 (Model 2). Significance was considered at a 2-sided *P* value of <.05. The observational, secondary data analysis of publicly available, de-identified data was deemed exempt by the Yale University Institutional Review Board.

## Results

Among adolescents who had never used e-cigarettes at Wave 4 (n = 7,872), 16.4% reported nondaily use and 65.9% daily use of social media at Wave 4 (Table 2). At Wave 5, 62.9% still did not use e-cigarettes and reported not being susceptible to e-cigarette use, while 17.6% did not use e-cigarettes but reported being susceptible to e-cigarette use. Also at Wave 5, 10.8% of adolescents had initiated e-cigarette use but had not used e-cigarettes in the past 30 days, and 8.7% had initiated e-cigarette use and had used e-cigarettes in the past 30 days (Table 2).

**Table 2:** Characteristics of Sample at Wave 4 (2016–2018) by E-Cigarette Use and Susceptibility^a^ Status at Wave 5 (2018–2019) Among Adolescents Who Never Used E-Cigarettes at Wave 4, PATH Study^b^

Predictors at Wave 4	Overall no. (weighted %)^c^	Outcome: E-cigarette use and susceptibility status at Wave 5, no. (weighted %)^c^				*P* value^f^
		Nonsusceptible/never e-cigarette use	Susceptible/never e-cigarette use	Past e-cigarette use^d^	Current e-cigarette use^e^	
**Overall no. (%)**	7,872 (100.0)	4,978 (62.9)	1,387 (17.6)	801 (10.8)	665 (8.7)	—
**Social media use**						
Never	1,389 (17.7)	1,062 (21.7)	200 (14.8)	61 (6.7)	48 (6.6)	<.001
Nondaily	1,327 (16.4)	933 (18.4)	224 (15.5)	100 (12.3)	66 (9.8)	
Daily	5,145 (65.9)	2,978 (59.9)	960 (69.6)	640 (81.0)	551 (83.7)	
**Age, y**						
12–14	6,084 (77.6)	3,901 (79.1)	1,089 (79.0)	586 (72.4)	471 (69.6)	<.001
15 or 16	1,788 (22.4)	1,077 (20.9)	298 (21.0)	215 (27.6)	194 (30.4)	
**Sex**						
Female	3,828 (49.6)	2,343 (48.3)	713 (51.8)	413 (52.3)	344 (51.7)	.01
Male	4,044 (50.4)	2,635 (51.7)	674 (48.2)	388 (47.7)	321 (48.3)	
**Hispanic**						
No	5,498 (76.3)	3,442 (75.6)	926 (74.1)	587 (79.9)	518 (82.8)	<.001
Yes	2,374 (23.7)	1,536 (24.4)	461 (25.9)	214 (20.1)	147 (17.2)	
**Race**						
White only	5,316 (69.6)	3,227 (66.2)	949 (70.8)	600 (77.2)	512 (81.8)	<.001
Black only	1,291 (15.3)	951 (18.1)	196 (13.6)	81 (9.2)	55 (6.7)	
Other^g^	1,265 (15.1)	800 (15.8)	242 (15.6)	120 (13.6)	98 (11.5)	
**Parental education**						
Less than high school	1,124 (11.9)	737 (12.2)	210 (12.7)	97 (10.0)	73 (9.8)	.11
GED	326 (3.7)	207 (3.6)	50 (3.4)	32 (3.7)	35 (5.4)	
High school graduate	1,348 (16.2)	886 (16.9)	225 (15.1)	126 (14.7)	106 (15.5)	
Some college (no degree) or associates degree	2,441 (30.7)	1,512 (30.2)	420 (29.8)	260 (31.5)	235 (34.8)	
Bachelor’s degree	1,555 (22.3)	980 (22.3)	277 (22.1)	156 (22.3)	133 (21.6)	
Advanced degree	1,013 (15.3)	615 (14.8)	193 (16.9)	126 (17.9)	78 (12.8)	
**Annual household income, $**						
<10,000	663 (7.5)	458 (8.3)	113 (7.4)	48 (5.0)	41 (5.7)	<.001
10,000–24,999	1,161 (13.4)	754 (13.8)	205 (13.4)	111 (12.1)	80 (11.0)	
25,000–49,999	1,743 (21.4)	1,128 (21.9)	281 (19.7)	165 (19.0)	162 (24.2)	
50,000–99,999	1,872 (26.1)	1,162 (25.9)	312 (24.3)	206 (27.3)	182 (29.4)	
≥100,000	2,048 (31.6)	1,214 (30.1)	403 (35.2)	248 (36.7)	177 (29.7)	
**Susceptibility to e-cigarette use at Wave 4**						
No	5,926 (83.1)	4,076 (92.9)	942 (73.7)	503 (66.5)	379 (57.8)	<.001
Yes	1,231 (16.9)	331 (7.1)	352 (26.3)	272 (33.5)	269 (42.2)	
**Parental e-cigarette use**						
No	7,565 (96.7)	4,819 (97.5)	1,330 (96.4)	757 (95.3)	622 (94.3)	<.001
Yes	252 (3.3)	124 (2.5)	48 (3.6)	40 (4.7)	39 (5.7)	
**Peer e-cigarette use**						
None	6,904 (88.3)	4,616 (93.4)	1,181 (86.5)	606 (76.0)	465 (70.7)	<.001
Any	920 (11.7)	336 (6.6)	196 (13.5)	188 (24.0)	196 (29.3)	
**Other tobacco use (eg, cigarettes, cigars, smokeless tobacco, hookah)**						
None	7,729 (99.3)	4,907 (99.7)	1,365 (99.6)	775 (98.4)	642 (97.1)	<.001
Any	54 (0.7)	13 (0.3)	8 (0.4)	15 (1.6)	18 (2.9)	
**Current alcohol use**						
No	7,565 (96.1)	4,871 (97.8)	1,326 (95.7)	736 (92.4)	592 (88.7)	<.001
Yes	304 (3.9)	106 (2.2)	60 (4.3)	64 (7.6)	73 (11.3)	
**Current cannabis use**						
No	7,789 (99.0)	4,950 (99.5)	1,374 (99.2)	777 (97.3)	648 (97.0)	<.001
Yes	83 (1.0)	28 (0.5)	13 (0.8)	24 (2.7)	17 (3.0)	
**Current any drug use (eg, misuse of prescribed drugs, illicit drugs)**						
No	7,642 (97.2)	4,862 (97.9)	1,343 (96.8)	764 (95.8)	633 (95.0)	<.001
Yes	228 (2.8)	115 (2.1)	43 (3.2)	37 (4.2)	32 (5.0)	

Abbreviation: GED, General Educational Development; PATH, Population Assessment of Tobacco and Health.

a Susceptibility was categorized on the basis of “intention to use” (“Do you think you might try using e-cigarettes soon?”) and “willingness of e-cigarette use” (“If one of your best friends were to offer you e-cigarettes, would you use it?”). Only when respondents reported “definitely not” to both questions were they categorized as “non-susceptible never-use”; others were categorized as “susceptible never-use.”

b Data source: Hyland et al (12).

c Values in each category may not add to totals because of missing values; percentages may not add to 100 because of rounding.

d Initiated e-cigarette use at Wave 5 but did not use e-cigarettes in the past 30 days.

e Initiated e-cigarette use at Wave 5 and still used e-cigarettes in the past 30 days.

f Rao–Scott adjusted χ^2^ test for categorical variables; Wald adjusted test for continuous variables.

g Includes American Indian or Alaska Native, Asian Indian, Asian, Native Hawaiian, and Pacific Islander.

### Association between social media and susceptibility to and initiation of e-cigarette use

Among adolescents who had never used e-cigarettes at Wave 4 (n = 7,872) (Model 1, Table 3), nondaily social media use (vs never) at Wave 4 was significantly associated with a higher likelihood of past e-cigarette use (adjusted odds ratio [aOR] = 2.26; 95% CI, 1.51–3.37) and current e-cigarette use at Wave 5 (aOR = 1.64; 95% CI, 1.04–2.60). Daily social media use (vs never) was significantly associated with a higher likelihood of being susceptible to e-cigarette use (aOR = 1.46; 95% CI, 1.20–1.78), past e-cigarette use (aOR = 3.55; 95% CI, 2.49–5.06), and current e-cigarette use (aOR = 3.45; 95% CI, 2.38–5.02) at Wave 5.

**Table 3:** Results of Multivariable Multinomial Logistic Regression (Model 1) on E-Cigarette Susceptibility^a^ and Use Behaviors at Wave 5 (2018–2019) Among Adolescents (n = 7,872) Who Never Used E-Cigarettes at Wave 4 (2016–2018), PATH Study^b^

Wave 4 social media use	Wave 5 e-cigarette susceptibility and use behaviors, adjusted OR (95% CI) [*P* value]^c^		
	Never used e-cigarettes, but susceptible to e-cigarette use (n = 1,387)	Past e-cigarette use (n = 801)^d^	Current e-cigarette use (n = 665)^e^
Never	1 [Reference]	1 [Reference]	1 [Reference]
Nondaily	1.17 (0.88–1.57) [.28]	2.26 (1.51–3.37) [<.001]	1.64 (1.04–2.60) [.03]
Daily	1.46 (1.20–1.78) [<.001]	3.55 (2.49–5.06) [<.001]	3.45 (2.38–5.02) [<.001]

Abbreviation: OR, odds ratio; PATH, Population Assessment of Tobacco and Health.

a Susceptibility was categorized on the basis of “intention to use” (“Do you think you might try using e-cigarettes soon?”) and “willingness of e-cigarette use” (“If one of your best friends were to offer you e-cigarettes, would you use it?”). Only when respondents reported “definitely not” to both questions were they categorized as “nonsusceptible never-use”; others were categorized as “susceptible never-use.”

b Data source: Hyland et al (12).

c The model was controlled for Wave 4 variables of age, sex, ethnicity, race, parental education, annual household income, parental and peer e-cigarette use, e-cigarette use susceptibility, current use of other tobacco products and substances. “Never used e-cigarettes and not susceptible to e-cigarette use” was used as reference group.

d Initiated e-cigarette use at Wave 5 but did not use e-cigarettes in the past 30 days.

e Initiated e-cigarette use at Wave 5 and still used e-cigarettes in the past 30 days.

### Associations between social media use and continued use of e-cigarettes

Among the 794 adolescents who ever used e-cigarettes at Wave 4 (Model 2, Table 4), 436 (52.9%, weighted) discontinued e-cigarette use at Wave 5, but 358 (47.1%, weighted) reported they still used e-cigarettes in the past 30 days at Wave 5. We found no significant association between continued e-cigarette use at Wave 5 and social media use at Wave 4 (all *P* values > .05).

**Table 4:** Results of Multivariable Binomial Logistic Regression (Model 2) on Continued Use of E-Cigarettes (Used E-Cigarettes in the Past 30 Days) at Wave 5 (2018–2019) Among Adolescents (n = 794) Who Ever Used E-Cigarettes at Wave 4 (2016–2018), PATH Study^a^

Wave 4 social media use	Continued use of e-cigarettes at Wave 5, adjusted OR (95% CI) [*P* value] (n = 358)^b^^,^ c
Never	1 [Reference]
Nondaily	1.74 (0.81–3.74) [.15]
Daily	1.80 (0.97–3.34) [.06]

Abbreviation: OR, odds ratio; PATH, Population Assessment of Tobacco and Health.

a Data source: Hyland et al (12).

b The model was controlled for Wave 4 variables of age, sex, ethnicity, race, parental education, annual household income, parental and peer e-cigarette use, current use of other tobacco products and substances. Noncurrent e-cigarette use at Wave 5 (ie, ever used e-cigarettes at Wave 4, but did not use e-cigarettes in the past 30 days at Wave 5), used as reference group.

c Ever used e-cigarettes at Wave 4 and still used e-cigarettes in the past 30 days at Wave 5.

## Discussion

We observed that social media use was associated with subsequent susceptibility to e-cigarette use and initiation but not with continued use of e-cigarettes among US adolescents aged 12 to 16 years. Susceptibility and e-cigarette initiation among adolescents may be driven by exposure to e-cigarette–related content on social media. Although we did not examine the content viewed by adolescents on social media, studies have documented pro–e-cigarette content and promotion on social media (3). Furthermore, previous studies identified reasons that adolescents experiment with e-cigarettes, including their use by peers and their various flavors (10,11), which are frequently reflected in social media e-cigarette content (17,18). We posit that exposure to such themes might be related to higher levels of susceptibility to and initiation of e-cigarette use among adolescents. Given the constantly changing social media environment and e-cigarette promotion on social media (4), future studies should monitor these associations as newer PATH data sets become available.

Unexpectedly, we did not find a significant association between continued use of e-cigarettes at Wave 5 and social media use at Wave 4. This finding might suggest that once an adolescent starts to use e-cigarettes, social media use may not contribute to the progression to continued e-cigarette use. This finding was somewhat unexpected because a previous study suggested that exposure among young adult college students to e-cigarette–related content on social media was associated with an increased level of e-cigarette use 1 year later (19). We speculate that once e-cigarette use is initiated, other factors, such as e-cigarette use dependence, might influence continued use more than social media. Future studies should examine factors associated with continued e-cigarette use among adolescents.

It is also important to consider other unmeasured psychological and environmental factors that may influence the relationship between social media use and e-cigarette use among adolescents. For example, lower levels of social media use might be associated with having parents who have a protective parenting style that may either restrict social media screen time or adolescent e-cigarette use (20). Furthermore, adolescents who do not use social media might be active in other extracurricular activities, which might protect against e-cigarette use (21). Future studies should examine the characteristics of adolescent survey respondents who do not use social media to understand protective factors that might contribute to preventing e-cigarette use.

### Limitations

Our study has several limitations. First, because our study was observational, we cannot assume a causal relationship between social media use and e-cigarette use behaviors. Second, we examined general social media use, so it was not possible to determine what kind of content was seen by respondents. Third, the PATH Study asked about general social media platforms; thus, we cannot determine which social media platforms PATH respondents visited. Because each social media platform has its own policies, unique characteristics (eg, video-based, text-based), and features (eg, retweets), future studies should examine the effects of these characteristics on e-cigarette use. Fourth, we did not test potential underlying mechanisms between social media use and e-cigarette use behaviors. However, previous studies observed that exposure to e-cigarette advertisements, risk perceptions of e-cigarettes, and e-cigarette expectancies mediated the association between social media use and e-cigarette use behaviors (16,19). Future studies should examine other potential mediators between social media use and e-cigarette use behaviors.

### Conclusion

Our study highlights the need for social media–related strategies that prevent e-cigarette use among adolescents. These strategies could include developing and disseminating counter-messaging (ie, anti–e-cigarette campaigns) on social media. Incorporating social media components in e-cigarette use prevention strategies tailored for adolescents may increase effectiveness and eventually reduce e-cigarette–related health consequences in underage populations. Continued surveillance and regulations on e-cigarette–related content on social media at tobacco regulatory agencies could also help to curb e-cigarette use among adolescents.
